# Spontaneous Bacterial Peritonitis: Etiology, Microbiology, and Clinical Outcomes in Cirrhosis Patients

**DOI:** 10.7759/cureus.76679

**Published:** 2024-12-31

**Authors:** Lal Krishna U, Deni J, Ramu M, Sandesh K, Saji K Sebastian, Gaurav Khatana, Gino R Philip

**Affiliations:** 1 Medical Gastroenterology, Government Medical College, Kottayam, IND; 2 Gastroenterology and Hepatology, Government Medical College, Kottayam, IND

**Keywords:** antibiotic sensitivity, ascites, drug resistance, liver disease, spontaneous bacterial peritonitis

## Abstract

Objective: Spontaneous bacterial peritonitis (SBP) is a critical complication in patients with liver cirrhosis, often resulting in high mortality. Understanding the microbiological agents causing SBP and their antibiotic resistance patterns is essential for effective treatment, particularly in tertiary care settings. This prospective observational study aimed to identify the microbial profile of SBP, evaluate antibiotic sensitivity, and assess patient outcomes.

Methodology: The study included 100 patients over 18 years old with chronic liver disease and SBP. Data collected included demographics, ascitic fluid analysis, cultures, liver and renal function tests, ultrasonograms, and disease etiology. Scoring systems such as sequential organ failure assessment (SOFA), child-turcotte-pugh (CTP), and model for end-stage liver disease (MELD) were calculated. Patients received standard care, and outcomes (discharge or mortality) were recorded.

Results: Of the 100 patients with SBP, 91% were men. Most were classified as child-turcotte-pugh Class C (66%), with the remainder as Class B (34%). The leading cause of cirrhosis was alcohol use (72%), followed by metabolic-associated steatotic liver disease (MASLD), hepatitis B virus (HBV), and hepatitis C virus (HCV). Prior antibiotic exposure was noted in 21% of cases. Despite prophylaxis, SBP developed in 19%. Ascitic fluid cultures showed no growth in 56%, but *Escherichia coli* (16%) and Klebsiella species (8%) were the most common pathogens isolated. Acute-on-chronic liver failure (ACLF) occurred in 19%, with a mortality rate of 89%. Multidrug-resistant (MDR) and extensively drug-resistant (XDR) pathogens were identified in 5% and 3% of cases, respectively.

Conclusion: This study identifies *Escherichia coli* as the most prevalent pathogen in SBP and highlights the impact of comorbidities like diabetes and dyslipidemia on outcomes. High sequential organ failure assessment scores, hepatic encephalopathy, variceal bleeding, renal failure, mechanical ventilation, and alcoholic liver disease significantly increased mortality risk. The emergence of multi-drug-resistant and extensively drug-resistant pathogens underscores the need for vigilant monitoring, early intervention, and customized antibiotic therapies to manage SBP effectively in cirrhotic patients.

## Introduction

Liver cirrhosis is a severe condition associated with multiple complications, one of the most serious being spontaneous bacterial peritonitis (SBP). This life-threatening infection occurs in patients with cirrhosis and ascites and is characterized by bacterial infection of ascitic fluid without an identifiable intra-abdominal source. Diagnostic criteria, such as an absolute neutrophil count (ANC)>250/mm³, are essential for early detection and timely treatment to prevent progression to septic shock [1].

Among infections in cirrhotic patients, SBP is the most common, with morbidity and mortality rates ranging from 20% to 40% [2]. Its complications include hepatic decompensation and renal impairment, both of which can further worsen patient outcomes. The primary pathogens involved are gram-negative bacteria, notably *Escherichia coli *and *Klebsiella pneumoniae*. However, rising antibiotic resistance has significantly challenged effective treatment. For instance, empirical therapy with third-generation cephalosporins shows failure rates between 33% and 75%, underscoring the need for antibiotic regimens tailored to local resistance patterns [3]. SBP is classified into three types: classic SBP, culture-negative neutrophilic ascites (CNNA), and monomicrobial non-neutrocytic bacterascites (MNB). While the prevalence of classic SBP has declined due to widespread antibiotic prophylaxis, CNNA has become more common, indicating a shift in infection dynamics [4]. The pathogenesis of SBP is complex and involves gut dysbiosis, bacterial translocation, and impaired host defense mechanisms, all of which contribute to infection risk.

Bacterial translocation from the gastrointestinal tract is the primary source of SBP, predominantly involving gram-negative bacilli (GNB). However, gram-positive cocci (GPC) have become increasingly prevalent due to invasive treatments and long-term quinolone use [5]. The emergence of multidrug-resistant (MDR) organisms has further complicated SBP bacteriology and treatment strategies. Enterobacteriaceae primarily causes community-acquired cases, whereas nosocomial cases frequently involve GPC and MDR organisms. Notably, nosocomial and staphylococcal infections are associated with higher mortality rates compared to community-acquired infections. Moreover, nosocomial infections are more likely to involve bacteria resistant to multiple drugs, such as extended-spectrum β-lactamase-producing enterobacteriaceae (ESBL-E) [6].

Gut dysbiosis, characterized by an overgrowth of *E. coli*, is considered an early step in SBP development. Liver cirrhosis exacerbates this disruption by impairing gut motility and reducing gastric acid levels, promoting bacterial overgrowth [7]. Increased intestinal permeability in cirrhotic patients further facilitates bacterial entry into the bloodstream, increasing the risk of infection [8]. The movement of bacteria from the gut to mesenteric lymph nodes, known as bacterial translocation, is a well-documented phenomenon in animal models of SBP [9]. From the lymph nodes, bacteria can travel to the bloodstream and eventually infiltrate the ascitic fluid. Studies have shown bacterial translocation occurs more frequently in patients with advanced cirrhosis [10]. Patients with cirrhosis and ascites often have bacterial DNA in their bodily fluids, even if bacteria cannot be grown in culture [11]. This indicates that even small amounts of bacteria or bacterial fragments may translocate from the gut, activating tumor necrosis factor (TNF) and heightening the risk of SBP [12]. Notably, bacterial DNA alone is associated with increased mortality in cirrhotic patients, emphasizing the significance of early intervention.

Effective management of SBP requires a comprehensive approach, including early diagnosis, timely and appropriate therapy, and ongoing monitoring. The rising issue of antibiotic resistance highlights the need for individualized treatment strategies informed by local microbiological data. By adopting a patient-centered, evidence-based approach and leveraging multidisciplinary collaboration, healthcare providers can improve outcomes and quality of life for patients facing this severe complication of liver cirrhosis. Recognizing these challenges, this study aimed to identify the microbial profile of SBP, evaluate antibiotic sensitivity, and assess patient outcomes.

## Materials and methods

This prospective observational study was conducted in the department of Gastroenterology at Government Medical College, Kottayam, over 12 months (from June 2023 to June 2024). The research took place within the department of Gastroenterology and the medical wards of the institution. The study population included patients aged 18 years or older diagnosed with spontaneous bacterial peritonitis (SBP) and admitted to these facilities.

The sample size was determined using data from a previous study by Ganesh Bhat et al. which reported a 50% sensitivity to ceftriaxone among culture-positive patients [1].

Using the formula

\[ \frac{4pq}{d^2} \]

\[ p = 50\%, \quad q = 100\% - p = 50\%, \quad d = 10\% \]

(allowable error), the calculated sample size was 100.

Inclusion and exclusion criteria

Participants included in the study were patients with a confirmed diagnosis of SBP, defined by an ascitic neutrophil count exceeding 250 cells/mm³ or a total leukocyte count greater than 500 cells/mm³ in the context of chronic liver disease. They were required to be at least 18 years old. Patients with pre-existing organ dysfunction (e.g., chronic kidney disease, heart failure), tuberculosis, malignancy, or pregnancy were excluded.

Ethical considerations

The Institutional Review Board of Government Medical College, Kottayam, approved the study. Written informed consent was obtained from all participants or their legal guardians before enrollment. Consecutive patients with cirrhosis and a confirmed diagnosis of SBP were included in the study. Detailed patient histories were collected to evaluate the status and underlying causes of chronic liver disease. Comprehensive clinical assessments, including general and systemic evaluations, were conducted. The severity of liver cirrhosis was assessed using the child-pugh classification and model for end-stage liver disease (MELD) scores.

Procedures

For all patients with suspected SBP, ascitic fluid analysis was performed, including culture and antibiotic sensitivity testing. The primary focus was to identify etiological agents causing SBP, evaluate their antibiotic sensitivity profiles, and assess the correlation between these factors and clinical outcomes. Special attention was given to the sensitivity of third-generation cephalosporins.

Data analysis

Data were systematically recorded in Microsoft Excel and analyzed using IBM SPSS software. Quantitative variables were reported as means and standard deviations, while qualitative variables were expressed as proportions. Associations between variables were evaluated using the chi-square test for qualitative data and the t-test for quantitative data. A p-value of less than 0.05 was considered statistically significant.

## Results

Table 1 shows the demographic and clinical characteristics of the study population, which consisted of 100 subjects with a mean age of 50.94±7.06 years. The majority of the participants were male (91%, n=91), with ascites (94, 94%) being the most common presenting complaint. Comorbidities were prevalent, with dyslipidemia (45, 45%) and diabetes (44, 44%) being the most common. A significant portion of patients (70%, n=70) had at least one comorbidity. The child-pugh classification indicated that 34% (n=34) of the subjects were classified as Class B, while the remaining 66 (66%) were Class C. The primary etiology was primarily ethanol use (72, 72%).

**Table 1:** Demographic and clinical characteristics of the study population.

Variables	Values N=100
Mean age	50.94±7.06
Gender distribution	
Male	91 (91%)
Female	9 (9%)
Complaints	
Ascites	94 (94%)
Abdominal pain	72 (72%)
Fever	54 (54%)
Comorbidities	
Dyslipidemia	45 (45%)
Diabetes	44 (44%)
Hypertension	11 (11%)
Patient with at least one comorbidity	70 (70%)
Child Pugh classification	
Class B	34 (34%)
Class C	66 (66%)
Mean MELD Na score	27.77±3.41
Mean SOFA score	2.62±1.11
Primary etiology	
Ethanol use	72 (72%)
MASLD	12 (12%)
Met ALD	10 (10%)

Table 2 presents the microbiological and antibiotic resistance patterns of bacterial species identified in the study. Resistance rates varied among the antibiotics tested, with cefuroxime showing a resistance rate of 65.5% (n=65), followed by ciprofloxacin at 61 (61%) and ceftriaxone at 61 (61.8%). Notably, the carbapenems (ertapenem, imipenem, and meropenem) displayed significantly lower resistance rates, with 4.5%, 5.7%, and 5.9%, respectively. This highlights the prevalence of antibiotic resistance among common pathogens in the study population.

**Table 2:** Microbiological and antibiotic resistance patterns. Resistance, sensitivity, and intermediate susceptibility rates for various antibiotics are expressed as percentages.

Bacterial species identified	Resistant (%)	Sensitive (%)	Intermediate susceptibility (%)
Ciprofloxacin	61%	36.60%	2.40%
Trimethoprim-sulfamethoxazole	45.50%	45.50%	9.10%
Ampicillin	50%	50%	-
Amoxicillin	35.70%	60.70%	3.60%
Piperacillin	41.70%	52.80%	5.60%
Cefuroxime	65.50%	31%	3.40%
Ceftriaxone	61.80%	35.30%	2.90%
Cefoperazone-sulbactam	43.30%	50%	6.70%
Cefipime	52.60%	42.10%	5.30%
Ceftazidime-avibactam	33.30%	33.30%	33.30%
Ertapenem	4.50%	95.50%	-
Imipenem	5.70%	94.30%	-
Meropenem	5.90%	91.20%	2.90%
Amikacin	33.30%	60.60%	6.10%
Gentamycin	53.10%	46.90%	-
Colistin	-	66.70%	33.30%

Table 3 summarizes the hospitalization and outcome data. The mean duration of hospital stay was 5.74 ± 1.41 days with a mortality rate of 19 (19%) and a survival rate of 81% (n=81). Complications such as hypotensive shock (21, 21%) and acute-on-chronic liver failure (19, 19%) were noted. Additionally, renal replacement therapy was required in 10 (10%) of cases, and 8 (8%) of patients needed mechanical ventilation during hospitalization.

**Table 3:** Hospitalization and outcome data. Mean duration of hospital stay is reported in days (mean ± SD, median [IQR]), along with mortality and complications (frequency, percentage).

Outcome and complications	Value
Mean duration of hospital stay (days)	5.74 ± 1.41
Median hospital stay (days)	5 (IQR 5-7)
Mortality rate	19 (19%)
Survival rate	81 (81%)
Hypotensive shock	21 (21%)
ACLF (as per APASL criteria)	19 (19%)
Renal replacement therapy	10 (10%)
Mechanical ventilation	8 (8%)
Prior history of spontaneous bacterial peritonitis (SBP)	17 (17%)
Norflox prophylaxis	19 (19%)

The statistical analysis revealed several factors significantly associated with mortality in patients. Among continuous variables, age (chi-square=1.51, p=0.042), SOFA score (chi-square=3.73, p<0.001), and MELD Na score (chi-square=-0.59, p=0.008) were significantly higher in deceased patients, indicating greater illness severity. For categorical variables, significant associations were observed with comorbidities such as diabetes (chi-square=9.94, p=0.001), dyslipidemia (chi-square=6.43, p=0.005), and the presence of multiple comorbidities (chi-square=5.46, p=0.009). Critical complications, including hepatic encephalopathy (chi-square=22.07, p<0.001), variceal bleeding (chi-square=24.08, p<0.001), and ACLF as defined by APASL (chi-square=7015, p<0.001) were strongly associated with mortality. Markers of critical illness, such as hypotension shock (chi-square=61.3, p<0.001), mechanical ventilation (chi-square=31.57, p<0.001), and renal replacement therapy (chi-square=41.7, p<0.001), were also significant, with higher mortality observed in patients experiencing these conditions. Conversely, factors such as sex (chi-square=0.0, p=1.0), fever (chi-square=0.4, p=0.525), ascites (chi-square=0.0, p=1.0), and hypertension (chi-square=1.32, p=0.213) showed no significant associations with mortality. These findings underscore the importance of managing comorbidities and complications, particularly those reflecting critical illness, to improve patient outcomes (Table 4).

**Table 4:** Factors associated with outcome. Data is presented as mean±SD or frequency (percentage). Statistical significance was determined using Chi-square tests or independent t-tests, with p-values provided for each factor. *: A p-value of <0.05 indicates statistical significance.

Factors	Outcome		Statistical test	p-value
	Death (N=19)	Survived (N=81)		
Age			1.51	0.042*
Mean±SD	53.89±9.11	50.25±6.36		
Sex			0.0	1
Female	2 (22.2%)	7 (77.8%)		
Male	17 (18.7%)	74 (81.3%)		
Fever			0.4	0.525
Yes	12 (22.2%)	42 (77.8%)		
No	7 (15.2%)	39 (84.8%)		
Abdominal pain			1.07	0.188
Yes	16 (22.2%)	56 (77.8%)		
No	3 (10.7%)	25 (89.3%)		
Ascites			0	1.000
Yes	18 (19.1%)	76 (80.9%)		
No	1 (16.7%)	5 (83.3%)		
Diabetes			9.94	0.001*
Yes	15 (34.1%)	29 (65.9%)		
No	4 (7.1%)	52 (92.9%)		
Dyslipidemia			6.43	0.005*
Yes	14 (31.1%)	31 (68.9%)		
No	5 (9.1%)	50 (90.9%)		
Hypertension			1.32	0.213
Yes	4 (36.4%)	7 (63.6%)		
No	15 (16.9%)	74 (83.1%)		
Hypothyroidism			0.85	0.174
Yes	3 (37.5%)	5 (62.5%)		
No	16 (17.4%)	76 (82.6%)		
CAD			1.37	0.124
Yes	3 (42.9%)	4 (57.1%)		
No	16 (17.2%)	77 (82.8%)		
Comorbidities			5.46	0.009*
Yes	18 (25.7%)	52 (74.3%)		
No	1 (3.3%)	29 (96.7%)		
Child status			1.11	0.186
B	4 (11.8%)	30 (88.2%)		
C	15 (22.7%)	51 (77.3%)		
SOFA score			3.73	<0.001*
Mean ± SD	4.26 ± 1.28	2.23 ± 0.61		
MELD Na score			-0.59	0.008*
Mean ± SD	29.63 ± 2.43	27.33 ± 3.47		
Etiology			6.79	0.236
Cryptogenic	1 (100%)	0 (0%)		
Ethanol	14 (19.4%)	58 (80.6%)		
Hepatitis B	0 (0%)	3 (100%)		
Hepatitis C	1 (50%)	1 (50%)		
MASLD	2 (16.7%)	10 (83.3%)		
Met ALD	1 (10%)	9 (90%)		
History of prior antibiotic exposure			4.83	0.024*
Yes	8 (38.1%)	13 (61.9%)		
No	11 (13.9%)	68 (86.1%)		
Cirrhosis decompensation events or complication			17.6	<0.001*
Yes	15 (42.9%)	20 (57.1%)		
No	4 (6.2%)	61 (93.8%)		
Hepatic encephalopathy			22.07	<0.001*
Yes	11 (61.1%)	7 (38.9%)		
No	8 (9.8%)	74 (90.2%)		
Hepatocellular malignancy			9.54	0.162
Yes	2 (50%)	2 (50%)		
No	17 (17.7%)	79 (82.3%)		
Hepatorenal syndrome			0.93	0.162
Yes	2 (50%)	2 (50%)		
No	17 (17.7%)	79 (82.3%)		
Variceal bleeding			24.08	<0.001*
Yes	12 (60%)	8 (40%)		
No	7 (8.8%)	73 (91.3%)		
Others			0.01	0.917
Yes	0 (0%)	3 (100%)		
No	19 (19.6%)	78 (80.4%)		
Prior history of systolic blood pressure			2.37	0.086
Yes	6 (35.3%)	11 (64.7%)		
No	13 (15.7%)	70 (84.3%)		
Norflox prophylaxis			3.53	0.047*
Yes	7 (36.8%)	12 (63.2%)		
No	12 (14.8%)	69 (85.2%)		
Complications			59.65	<0.001*
Yes	18 (75%)	6 (25%)		
No	1 (1.3%)	75 (98.7%)		
ACLF definition APASL			7015	<0.001*
Yes	17 (89.5%)	2 (10.5%)		
No	2 (2.5%)	79 (97.5%)		
Hypotension shock			61.3	<0.001*
Yes	17 (81%)	4 (19%)		
No	2 (2.5%)	77 (97.5%)		
Mechanical ventilation			31.57	<0.001*
Yes	8 (100%)	0 (0%)		
No	11 (12%)	81 (88%)		
Renal replacement therapy			41.7	<0.001*
Yes	10 (100%)	0 (0%)		
No	9 (10%)	81 (90%)		
Duration of hospital stay			2.81	0.109
Mean±SD	6.21±2.48	5.63±1.01		

In Table 5, the analysis of bacterial organisms and outcomes revealed that *Acinetobacter baumannii* was significantly associated with mortality (chi-square=18.91, p=0.026), with 1 (50%) of patients dying and 1 (50%) surviving. *Enterococcus faecalis* was present in 3 (75%) of deceased patients and 1 (25%) of survivors, while *Escherichia coli *was found in 3 (18.8%) of deceased patients and 13 (81.2%) of survivors. *Klebsiella pneumoniae* was evenly distributed between deceased (4, 50%) and surviving patients (4, 50%). *Streptococcus alactolyticus* was identified in 1 (33.3%) of deceased patients and 2 (66.7%) of survivors. Cultures showing sterile/no growth were observed in 7 (12.5%) of deceased patients and 49 (87.5%) of survivors, suggesting a lower association with mortality. Other organisms, such as *Enterobacter cloacae* (0 deceased, 1, 100% survivors), *Klebsiella oxytoca *(0 deceased, 4, 100% survivors), Pseudomonas aeruginosa (0 deceased, 3, 100% survivors), and *Pseudomonas putida* (0 deceased, 3, 100% survivors), were exclusively found in survivors, indicating no association with mortality in this dataset.

**Table 5:** Analysis of bacterial organisms and outcomes. *: A p-value of <0.05 indicates statistical significance.

Organism	Outcome		Chi-square	p-value
	Death (N=19)	Survived (N=81)		
Acinetobacter baumanni	1 (50%)	1 (50%)	18.91	0.026*
Enterobacter cloacae	0 (0%)	1 (100%)		
Enterococcus feacalis	3 (75%)	1 (25%)		
Escherichia coli	3 (18.8%)	13 (81.2%)		
Klebsiella oxytoca	0 (0%)	4 (100%)		
Klebsiella pneumoniae	4 (50%)	4 (50%)		
Pseudomonas aeruginosa	0 (0%)	3 (100%)		
Pseudomonas putida	0 (0%)	3 (100%)		
Streptococcus alactolyticus	1 (33.3%)	2 (66.7%)		
Sterile / No growth	7 (12.5%)	49 (87.5%)		

The analysis of antimicrobial resistance factors and their association with outcomes revealed significant findings for specific resistance patterns in Table 6. MDR organism (chi-square=6.82, p=0.033) was significantly associated with mortality, with 3 (60%) of patients with MDR organisms dying, compared to 2 (40%) surviving. Patients without MDR organisms had lower mortality, with 7 (19.4%) deaths and 29 (80.6%) survivals. XDR organism (chi-square=14.4, p=0.001) also showed a strong association with mortality, as all patients (3, 100%) with XDR organisms died, whereas no patients with XDR organisms survived. In contrast, sterile cultures were associated with better outcomes, with 7 (13.2%) deaths and 46 (86.8%) survivals. Other factors, such as PDR (chi-square=1.42, p=0.143) and MDR with possible XDR (chi-square=3.41, p=0.181), did not show statistically significant associations. For PDR, 10 (23.8%) of patients without PDR died, while 32 (76.2%) survived. Among sterile cases, 7 (12.5%) died, while 49 (87.5%) survived. For MDR with possible XDR, 3 (21.4%) of patients died, while 11 (78.6%) survived in the group with resistance, but the association was not statistically significant. Finally, VRE (chi-square=0.55, p=0.214) was not significantly associated with outcomes, with 2 (28.6%) deaths and 5 (71.4%) survivals among patients with VRE. Sterile cultures showed a better prognosis, with 4 (10%) deaths and 36 (90%) survivals. These findings highlight the significant impact of MDR and XDR organisms on mortality while indicating limited or no association for other resistance patterns in this dataset.

**Table 6:** Effect of antimicrobial resistance factors with outcomes. PDR: pan-drug resistance, MDR: multi-drug resistance, XDR: extensively drug-resistant, VRE: vancomycin-resistant enterococcus. *: A p-value of <0.05 indicates statistical significance.

Factors	Outcome		Chi-square	p-value
	Death (N=19)	Survived (N=81)		
PDR			1.42	0.143
Sterile	7 (12.5%)	49 (87.5%)		
No	10 (23.8%)	32 (76.2%)		
MDR with possible XDR			3.41	0.181
Sterile	7 (12.3%)	50 (87.7%)		
No	7 (29.2%)	17 (70.8%)		
Yes	3 (21.4%)	11 (78.6%)		
MDR organism			6.82	0.033*
Sterile	7 (13.2%)	46 (86.8%)		
No	7 (19.4%)	29 (80.6%)		
Yes	3 (60%)	2 (40%)		
XDR organism			14.4	0.001*
Sterile	7 (13.2%)	46 (86.8%)		
No	7 (18.4%)	31 (81.6%)		
Yes	3 (100%)	0 (0%)		
VRE			0.55	0.214
Sterile	4 (10%)	36 (90%)		
No	2 (28.6%)	5 (71.4%)		

The analysis of antimicrobial agents and their outcomes revealed several significant associations. Ciprofloxacin was significantly associated with mortality (chi-square=9.62, p=0.008), with 11 (44%) of resistant cases resulting in death compared to 14 (56%) surviving. No deaths were observed among sensitive or intermediate cases. Trimethoprim-sulfamethoxazole also showed a significant association (chi-square 6.11, p=0.047), with 7 (46.7%) of resistant cases resulting in death, compared to 8 (53.3%) surviving. Among sensitive cases, 1 (6.7%) died, and 14 (93.3%) survived, while intermediate cases had 1 (33.3%) death and 2 (66.7%) survivals. Piperacillin was significantly associated with outcomes (chi-square=11.04, p=0.004), with 8 (53.3%) of resistant cases resulting in death compared to 7 (46.7%) surviving. Among sensitive cases, 1 (5.3%) died, and 18 (94.7%) survived. Cefoperazone-sulbactam also showed significant associations (chi-square=7.64, p=0.022), with 5 (38.5%) resistant cases resulting in death compared to 8 (61.5%) surviving. Among sensitive cases, no deaths were recorded, and all 15 (100%) survived. Intermediate cases had equal distributions, with 1 (50%) death and 1 (50%) survival. For gentamycin, a significant association was observed (chi-square=3.39, p = 0.041), with 7 (41.2%) resistant cases resulting in death compared to 10 (58.8%) surviving. Among sensitive cases, 1 (6.7%) died, and 14 (93.3%) survived. Other antibiotics, such as ampicillin (chi-square=0.0, p=1.000), tigecycline (chi-square=0.0, p=1.000), and benzylpenicillin (chi-square=0.0, p=1.000), showed no significant associations. Similarly, colistin (chi-square=0.47, p=0.792) and levofloxacin (chi-square=4.95, p=0.084) were not significantly associated with mortality, despite some differences in outcomes between resistant and sensitive cases. These findings highlight the impact of antimicrobial resistance, with significant associations observed for drugs like ciprofloxacin, trimethoprim-sulfamethoxazole, piperacillin, and gentamycin, while others showed limited or no impact on patient outcomes (Table 7).

**Table 7:** Association of antimicrobial resistance patterns with patient outcomes. *: A p-value of <0.05 indicates statistical significance.

Factors	Outcome		Chi-square	p-value
	Death (N=19)	Survived (N=81)		
Ciprofloxacin			9.62	0.008*
Resistant	11 (44%)	14 (56%)		
Sensitive	0 (0%)	15 (100%)		
Intermediate	0 (0%)	1 (100%)		
Trimethoprim -sulfamethoxazole			6.11	0.047*
Resistant	7 (46.7%)	8 (53.3%)		
Sensitive	1 (6.7%)	14 (93.3%)		
Intermediate	1 (33.3%)	2 (66.7%)		
Ampicillin			0	1.000
Resistant	4 (28.6%)	10 (71.4%)		
Sensitive	3 (21.4%)	11 (78.6%)		
Amoxicillin			2.02	0.364
Resistant	4 (40%)	6 (60%)		
Sensitive	3 (17.6%)	14 (82.4%)		
Intermediate	0 (0%)	1 (100%)		
Piperacillin			11.04	0.004*
Resistant	8 (53.3%)	7 (46.7%)		
Sensitive	1 (5.3%)	18 (94.7%)		
Intermediate	0 (0%)	2 (100%)		
Cefuroxime			4.86	0.088
Resistant	7 (36.8%)	12 (63.2%)		
Sensitive	0 (0%)	9 (100%)		
Intermediate	0 (0%)	1 (100%)		
Ceftriaxone			3.85	0.146
Resistant	8 (38.1%)	13 (61.9%)		
Sensitive	1 (8.3%)	11 (91.7%)		
Intermediate	0 (0%)	1 (100%)		
Cefoperazone sulbactam			7.64	0.022*
Resistant	5 (38.5%)	8 (61.5%)		
Sensitive	0 (0%)	15 (100%)		
Intermediate	1 (50%)	1 (50%)		
Cefipime			3.21	0.201
Resistant	3 (30%)	7 (70%)		
Sensitive	0 (0%)	8 (100%)		
Intermediate	0 (0%)	1 (100%)		
Ceftazidime avibactam			3.0	0.223
Resistant	0 (0%)	1 (100%)		
Sensitive	1 (100%)	0 (0%)		
Intermediate	0 (0%)	1 (100%)		
Imipenem			2.7	0.061
Resistant	2 (100%)	0 (0%)		
Sensitive	7 (21.2%)	26 (78.8%)		
Meropenem			7.11	0.061
Resistant	2 (100%)	0 (0%)		
Sensitive	6 (19.4%)	25 (80.6%)		
Intermediate	0 (0%)	1 (100%)		
Amikacin			4.01	0.135
Resistant	4 (36.4%)	7 (63.6%)		
Sensitive	2 (10%)	18 (90%)		
Intermediate	1 (50%)	1 (50%)		
Gentamycin			3.39	0.041*
Resistant	7 (41.2%)	10 (58.8%)		
Sensitive	1 (6.7%)	14 (93.3%)		
Tigecycline			0	1.000
Sensitive	2 (100%)	0 (0%)		
Intermediate	1 (50%)	1 (50%)		
Colistin			0.47	0.792
Resistant	1 (100%)	0 (0%)		
Sensitive	2 (66.7%)	1 (33.3%)		
Intermediate	2 (66.7%)	1 (33.3%)		
Benzyl penicillin			0	1.000
Resistant	2 (66.7%)	1 (33.3%)		
Sensitive	1 (33.3%)	2 (66.7%)		
Levofloxacin			4.95	0.084
Resistant	4 (57.1%)	3 (42.9%)		
Sensitive	0 (0%)	4 (100%)		
Intermediate	0 (0%)	2 (100%)		
Erythromycin			0	1.000
Resistant	2 (66.7%)	1 (33.3%)		
Sensitive	1 (33.3%)	2 (66.7%)		
Aztreonam			0	1.000
Resistant	1 (25%)	3 (75%)		
Sensitive	0 (0%)	2 (100%)		

## Discussion

Spontaneous bacterial peritonitis (SBP) is a severe complication in patients with cirrhosis, contributing significantly to morbidity and mortality. Understanding the factors influencing SBP outcomes-such as patient demographics, comorbidities, disease severity, and antibiotic resistance patterns, is critical for optimizing clinical management. This discussion evaluates the study’s findings, compares them with existing literature, highlights clinical implications, and outlines directions for future research.

The study reaffirms that older age is a pivotal factor influencing SBP outcomes. Older patients demonstrated higher mortality rates, consistent with findings by Zorreti et al. and Gambino et al., who reported greater disease severity, delayed diagnoses, and impaired immune responses in this population [13,14]. Fernandez et al. similarly emphasized that delayed diagnosis and severe disease presentations are more prevalent in older individuals, compounding their risk of poor outcomes [15].

Comorbidities, such as diabetes (p=0.001) and dyslipidemia (p=0.005), were significant predictors of mortality. Among participants, 70% had at least one comorbidity, which was strongly associated with increased mortality risk (p=0.009). These results align with studies by Tandon et al. [16] and Kimmann et al. [17], underscoring the role of comorbidities in exacerbating immune dysregulation and metabolic dysfunction in patients with cirrhosis.

The utility of disease severity scores in risk stratification was highlighted, with higher SOFA (p<0.001) and MELD-Na (p=0.008) scores significantly associated with increased mortality. These findings corroborate those of Peng et al. [18] and Tang et al. [19], who demonstrated the predictive value of these scoring systems in guiding SBP management and clinical decisions.

A notable finding of this study was the prevalence of multidrug-resistant organisms (MDROs) in SBP. Resistance was identified in 42.9% of cultures, with 14.7% harboring MDROs or extensively drug-resistant (XDR) organisms. Vancomycin-resistant enterococci (VRE) were present in 14.9% of cases. Ceftriaxone resistance, detected in 61.8% of cases with only 35.3% sensitivity, was particularly concerning. These results align with global trends reported by Fernandez et al. [15], Sarwar et al. [20], and Abd-elsalam et al. [21], where resistance rates reached up to 100% in nosocomial infections compared to community-acquired cases.

The emergence of ceftriaxone resistance is primarily attributed to the production of extended-spectrum beta-lactamases (ESBLs) and alterations in bacterial cell wall permeability, as reported by Khattak [22]. Gram-negative bacteria, particularly *E. coli and Klebsiella pneumoniae*, predominated in ascitic fluid infections and exhibited high resistance rates. This resistance undermines the efficacy of standard empirical therapies, necessitating broader-spectrum antibiotics such as carbapenems [23-24]. Although carbapenems and aminoglycosides demonstrated high sensitivity rates in this study, their use raises concerns about accelerating resistance development, emphasizing the need for robust antibiotic stewardship programs.

Ciprofloxacin resistance was observed in 61% of cases, further limiting treatment options. However, carbapenems (e.g., ertapenem, imipenem, meropenem) and aminoglycosides (e.g., amikacin, gentamicin) retained high sensitivity against *E. coli* and *K. pneumoniae*. These findings underscore the importance of individualized antibiotic regimens tailored to local resistance profiles to optimize outcomes and mitigate resistance.

Strengths and limitations

This study provides valuable insights into the determinants of SBP outcomes, emphasizing the importance of patient demographics, comorbidities, and disease severity in guiding management strategies. Early detection and aggressive treatment are essential for improving survival in high-risk populations. The observed antibiotic resistance patterns highlight the urgent need for antimicrobial stewardship and adherence to prescribing guidelines. However, the study’s limitations include a single-center design and a relatively small sample size, which may affect the generalizability of findings. Additionally, the absence of long-term follow-up data limits the assessment of outcomes beyond the acute management phase.

Future directions

Future research should focus on novel therapeutic approaches to address antibiotic resistance in SBP. Regional surveillance of resistance patterns, combined with evaluations of antimicrobial stewardship programs, is essential for sustainable management strategies. Advances in rapid diagnostic tools could facilitate the early identification of resistant organisms, enabling timely and targeted treatment interventions.

## Conclusions

In conclusion, this study provides important insights into the factors that influence outcomes in spontaneous bacterial peritonitis (SBP), including the role of disease severity, treatment response, and antibiotic resistance. By comparing its findings with previous studies and identifying areas for further research, the study highlights the need for a multidisciplinary and evidence-based approach to managing SBP. The growing problem of antibiotic resistance remains a severe challenge, making it essential to establish antimicrobial solid stewardship programs supported by ongoing monitoring of resistance patterns. These efforts are necessary to improve treatment strategies and patient outcomes. Further research is needed to refine methods for assessing risk, develop new treatment options, and address the global issue of antibiotic resistance. This study emphasizes the importance of a personalized approach to SBP management, focusing on early detection, tailored treatments, and strategies to reduce resistance. Continued collaboration and research will play a key role in advancing the understanding and care of SBP, ultimately improving patient outcomes and easing the burden on healthcare systems.
